# Effects of Hydroponic Barley Green Fodder Supplementation on Egg Quality and Proximate Composition in ATAK-S, Lohmann Brown, and Leghorn Laying Hens

**DOI:** 10.1021/acsomega.6c10822

**Published:** 2026-10-20

**Authors:** Süleyman Hacısalihoğlu, Samiye Adal, Zeynep Nur Karakuş, Yasemin Çelebi, Gülşah Çalışkan Koç

**Affiliations:** † Uşak University, Eşme Vocational School, Laborant and Veterinary Health, Eşme, Uşak 64200, Türkiye; ‡ Department of Food Engineering, Faculty of Engineering, 53002 Afyon Kocatepe University, Afyonkarahisar 03200, Türkiye; § Food Control, Application and Research Center, Afyon Kocatepe University, Afyonkarahisar 03200, Türkiye; ∥ Uşak University, Eşme Vocational School, Food Processing Department, Food Technology Program, Eşme, Uşak 64200 Türkiye

## Abstract

Hydroponic barley green fodder (HBGF) is increasingly discussed as a low-input, locally producible feed resource for poultry. This study examined how HBGF supplementation affects egg quality traits and the proximate composition of egg white and yolk in three laying-hen genotypes (ATAK-S, Lohmann Brown, and Leghorn) over a 42 day feeding period under free-range conditions. Hens of each genotype were allocated to a control group fed a commercial layer diet and to a group receiving the same feed plus fresh HBGF ad libitum. Egg weight, yolk, albumen, and eggshell percentages, eggshell thickness, egg shape index, yolk color, yolk index, albumen index, and Haugh unit were recorded on days 0, 21, and 42, and the dry matter, crude protein, crude fat, and ash contents of egg white and yolk were determined. Genotype was the primary contributor to variation in egg weight, egg shape index, and albumen index on every sampling day, in Haugh unit on days 0 and 21, in yolk percentage from day 21, and in albumen percentage on day 42 (*p* < 0.05–0.001). ATAK-S hens produced the heaviest eggs, Lohmann Brown hens had the greatest albumen percentage and egg shape index, and both ATAK-S and Leghorn hens had higher yolk percentage compared to Lohmann Brown. Albumen index and Haugh unit were higher in Lohmann Brown and Leghorn compared to ATAK-S. Eggshell thickness, eggshell percentage, yolk color, and yolk index did not differ among genotypes during the feeding period, apart from a genotype effect on yolk index on day 21 only. HBGF supplementation did not change any physical egg quality trait, including yolk color and yolk index, on day 21 or day 42 (*p* > 0.05). The proximate composition was mainly affected by the egg percentages (white versus yolk), with the yolk possessing significantly higher levels of dry matter, protein, fat, and ash contents compared to the white. HBGF produced compositional alterations that were both genotype and time dependent, with elevated albumen protein in ATAK-S and Lohmann Brown on day 21, increased yolk fat in ATAK-S and Lohmann Brown but decreased yolk fat in Leghorn, and no consistent change in albumen ash; however, these alterations varied among genotypes. Overall, genotype rather than HBGF influenced egg quality; however, HBGF produced slight changes in some compositional characteristics which tend to be genotype-specific rather than general improvements in egg quality.

## Graphical abstract

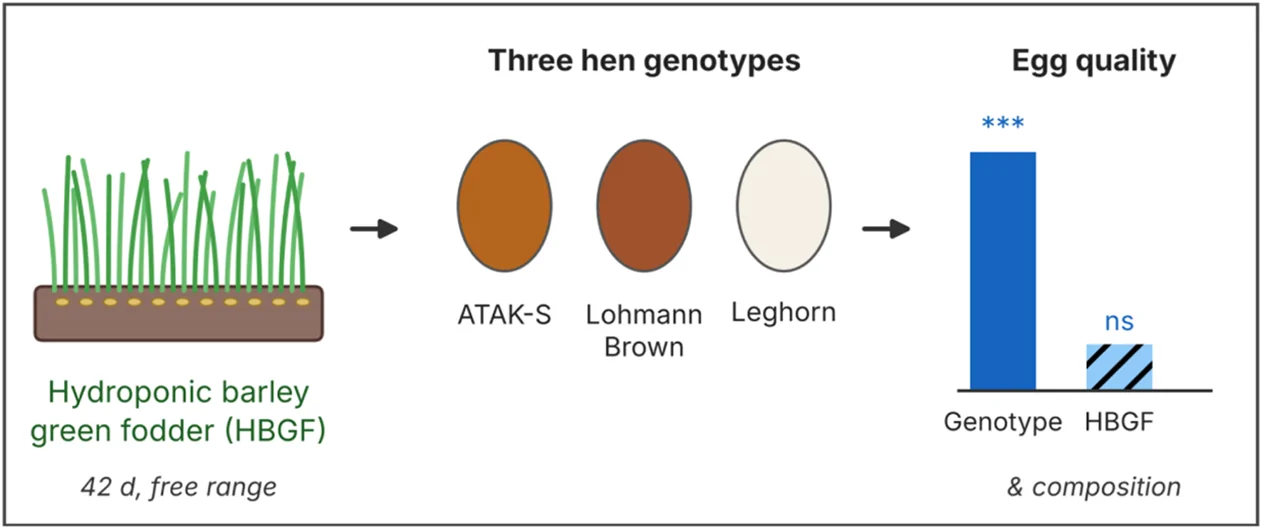

## 1 Introduction

Rising feed prices, pressure on arable land, and the sustainability targets set for livestock production have renewed interest in alternative feed resources for poultry.^1^ Sprouted cereals grown in hydroponic systems are one such resource. Barley, wheat, or maize grains are soaked, germinated, and grown for 6–10 days on trays with light and water only, yielding a green mat of shoots, roots, and residual grain that can be harvested within about a week regardless of season or soil.^2–4^ Germination reshapes the nutrient profile of the grain: crude protein, soluble sugars, cellulose, and ether extract increase, starch and β-glucan decrease, and water-soluble vitamins and phenolic compounds increase,^5,6^ while the green shoots additionally contribute chlorophyll and carotenoids.^7,8^ Sprouted barley is therefore best regarded as a fresh, moist green feed with a moderate protein concentration (roughly 12–20% of dry matter, depending on the crop and growing conditions) but a very low dry-matter content of around 10–15% as fed,^2,9^ so its contribution to the daily nutrient supply of a bird that already receives a complete diet is modest. Microgreens, the young seedlings of vegetables and cereals harvested 7–21 days after sowing, share many of these features and are reported to contain higher concentrations of phytochemicals, minerals, and vitamins than mature plants;^7,8^ feeding maize microgreens to chickens has recently been reported to improve growth.^10^

Studies on sprouted grains in laying hens is still limited. In laying Japanese quail, fresh hydroponic barley sprouts offered alongside a complete feed increased laying rate and fertility without changing the standard egg quality traits.^11^ In Lohmann Brown hens, a sprouted barley supplement of up to 45 g/day for 6 weeks did not affect body weight, lay rate, egg weight, or feed conversion, and the only measurable change was transient reductions in eggshell breaking strength in some supplemented groups (15 and 45 g/day on day 21 and 15 g/day on day 42).^12^ In ruminants, hydroponic barley has been associated with improved nutrient digestibility^13^ and, when fed with high-protein feeds, with a higher milk yield in dairy cows,^14^ although the very different digestive physiology of ruminants limits the transfer of these findings to poultry. More broadly, the responses of poultry to feed additives and byproducts, dried fruit pomace,^1,15^ apple pomace combined with a multienzyme preparation,^16^ fava beans,^17^ fermented *Broussonetia papyrifera*,^18^ spirulina with enzymes,^19^ curcumin, zinc nanoparticles, and *Bacillus licheniformis*,^20^ *Moringa oleifera* leaf meal,^21^ dietary fat sources,^22^ trace minerals such as zinc,^23^ and boron^24^, depend on the ingredient, its inclusion level, and the composition of the basal diet, and can extend from laying performance and egg quality to immune function.^15,25^ For example, Sosnówka-Czajka and Skomorucha^15^ found that dried raspberry and black currant pomace at 30 g/kg paled the yolk, that raspberry pomace also reduced egg weight and shell density in Hy-Line hens, and that black chokeberry and black currant pomace improved the white blood cell profile, while spleen weight increased with black currant and raspberry pomace.

Genotype is another major determinant of egg quality. Egg weight, shell thickness, albumen height, and Haugh unit have moderate to high heritabilities (roughly 0.3–0.6),^26^ and commercial brown- and white-egg hybrids differ from each other^27^ and from local or purebred genotypes^28,29^ in egg size, shell traits, and albumen quality. ATAK-S is a brown-egg layer hybrid developed by the Ankara Poultry Research Institute in Türkiye.^30^ Under free-range conditions, it has been reported to lay heavier eggs with thicker and stronger shells than the white-egg Turkish genotype Atabey, but with a lower yolk index, albumen index, and Haugh unit,^31^ and to differ from Lohmann Brown and Lohmann Sandy in laying rate, egg weight, shell strength, and yolk color.^32^ Whether the response of egg quality to a green fodder supplement differs among genotypes has not been examined, although genotype ×feed interactions are plausible given the differences in nutrient partitioning between hybrids selected for different purposes.

The aim of the present study was therefore to evaluate the effects of ad libitum HBGF supplementation of a commercial layer feed on the physical egg quality traits and the proximate composition of egg white and yolk in ATAK-S, Lohmann Brown, and Leghorn hens kept under free-range conditions and to test whether genotype modifies these responses over a 42-day feeding period.

## 2 Materials and Methods

### 2.1 Ethical Approval

The study protocol was reviewed by the Local Ethics Committee for Animal Experiments of Uşak University, Uşak, Türkiye, which concluded that formal ethical approval was not required under the institutional guidelines and regulations (07.05.2025; document no. E-72656843–050.04–279242).

### 2.2 Materials

Barley grain, commercial laying-hen feed, and hens were obtained from local suppliers in Eşme, Uşak, Türkiye.

### 2.3 Production of Hydroponic Barley Green Fodder and Feeding of the Hens

HBGF was produced from barley grain following the procedure described by Abouelezz et al.^11^ with minor modifications. Grains were soaked for 24 h (day 1), germinated in darkness for 2 days (days 2–3) and then grown on trays under controlled light and irrigation for a further 5 days (days 4–8), giving a total production period of 8 days, by which time the sprouts had reached approximately 15 cm. The feeding trial followed the design described in our previous study.^33^ Briefly, 72 laying hens of three genotypes (ATAK-S, Lohmann Brown, and Leghorn; 24 hens per genotype) were randomly allocated within genotype to a control and an HBGF group, each consisting of four replicate subgroups of three hens (Table 1). After a 7-day adaptation period, the hens were kept for 42 days under free-range conditions. Control groups received the commercial layer feed only, whereas HBGF groups received the same feed plus freshly harvested HBGF offered ad libitum. Further details of the experimental design, housing and feeding are given in our previous study.^33^

**Table 1:** Distribution of the Laying Hens Used in the Study

Egg-Laying Chicken Breed	Groups	Subgroups	Number of Chickens
ATAK-S	Control	Control 1	3
		Control 2	3
		Control 3	3
		Control 4	3
	Experimental	Experimental 1	3
		Experimental 2	3
		Experimental 3	3
		Experimental 4	3
LOHMANN BROWN	Control	Control 1	3
		Control 2	3
		Control 3	3
		Control 4	3
	Experimental	Experimental 1	3
		Experimental 2	3
		Experimental 3	3
		Experimental 4	3
LEGHORN	Control	Control 1	3
		Control 2	3
		Control 3	3
		Control 4	3
	Experimental	Experimental 1	3
		Experimental 2	3
		Experimental 3	3
		Experimental 4	3
TOTAL	6	24	72

### 2.4 Egg Analyses

#### 2.4.1 Egg Sample Preparation

Eggs were weighed on a digital balance (Shimadzu UX4200H, Shimadzu Corp., Kyoto, Japonya) with a precision of 0.01 g,^34^ and the long and short diameters of each egg were measured with a digital calliper (0.01 mm, Mitutoyo 500-196-30, Mitutoyo Corp., Kawasaki, Japan). Each egg was then broken onto a flat white surface and left for 5 min before yolk and albumen (white) heights and diameters were measured. The yolk was separated from the albumen and the weight was measured. The shells were rinsed under gently running tap water to remove adhering albumen, air-dried, and weighed.^35^ Eggs were analyzed on days 0, 21, and 42 of the feeding period.

#### 2.4.2 Physical Analyses

##### 2.4.2.1 Egg Content Parameters (EC)

Egg weight (EW) was recorded, and yolk and shell weights were measured after breaking; albumen weight was obtained by subtracting yolk and shell weights from the egg weight (Shimadzu UX4200H, Shimadzu Corp., Kyoto, Japonya). Yolk (YP), albumen (AP), and eggshell (EP) percentages were calculated relative to egg weight.^36^

##### 2.4.2.2 Exterior Egg Quality Parameters (EEQP)

Egg length and width were measured with a digital calliper (Mitutoyo 500-196-30, Mitutoyo Corp., Kawasaki, Japan), and the egg shape index (ESI) was calculated as width/length × 100.^36^ Eggshell thickness (ET) was measured with a digital calliper.

##### 2.4.2.3 Interior Egg Quality Parameters (IEQP)

Yolk color (YC) was scored with the Roche Yolk Color Fan (1–15). Yolk index (YI; yolk height/yolk diameter × 100), albumen index (AI; albumen height/mean albumen diameter × 100), and Haugh unit (HU = 100 log­(*H* – 1.7*W* ^0.37^ + 7.6), where *H* is albumen height in mm and *W* is egg weight in g) were calculated as described by Haugh^37^ and Sauveur and de Reviers.^36^

#### 2.4.3 Proximate Composition Analysis

Egg yolk and egg white were separated and analyzed individually. Dry matter content was determined gravimetrically by oven-drying at 105 °C to constant weight (Nüve FN 500, Nüve Industrial Materials Manufacturing and Trade Inc., Türkiye). Crude protein content was determined by the Kjeldahl method using a nitrogen-to-protein conversion factor of 6.25. Crude fat content was determined in the yolk only, by Soxhlet extraction with petroleum ether/diethyl ether. Ash content was determined by incineration of the dried samples in a muffle furnace at 550 °C until a constant, light-gray ash was obtained (Protherm PLF 110/6, Alser Teknik Seramik Inc., Türkiye). All analyses were carried out in triplicate according to AOAC.^38^

### 2.5 Statistical Analysis

The replicate subgroup (three hens) was the experimental unit; means and standard deviations (SD) were calculated from the four subgroup means of each genotype × feed combination (*n* = 4). For each sampling day (0, 21, and 42), the egg quality traits were analyzed by two-way analysis of variance (ANOVA) with genotype (G: ATAK-S, Lohmann Brown, Leghorn), feed (T: control, HBGF), and their interaction (G × T) as fixed effects. Significance levels are indicated as *p < 0.05, **p < 0.01, and ***p < 0.001; ns, not significant (*p* > 0.05). Proximate composition data were analyzed within each egg percentage (white or yolk) by one-way ANOVA among the three genotype × feed groups on each sampling day and among the three sampling days within each group, followed by Duncan’s test. Results are presented as mean ± SD. All statistical analyses were performed using IBM SPSS Statistics version 20.0 (IBM Corp., Armonk, NY, USA).

## 3 Results and Discussion

Feeding effects are assessed on days 21 and 42, whereas differences observed on day 0 reflect initial group heterogeneity rather than fodder effects, as samples were collected before dietary divergence. Day 0 results are given in Table S1, and the day 21 and day 42 results in Supplementary Tables S2 and S3; all three sampling days, with the Duncan letters, are shown in Figures 1–3.

**Figure 1:**
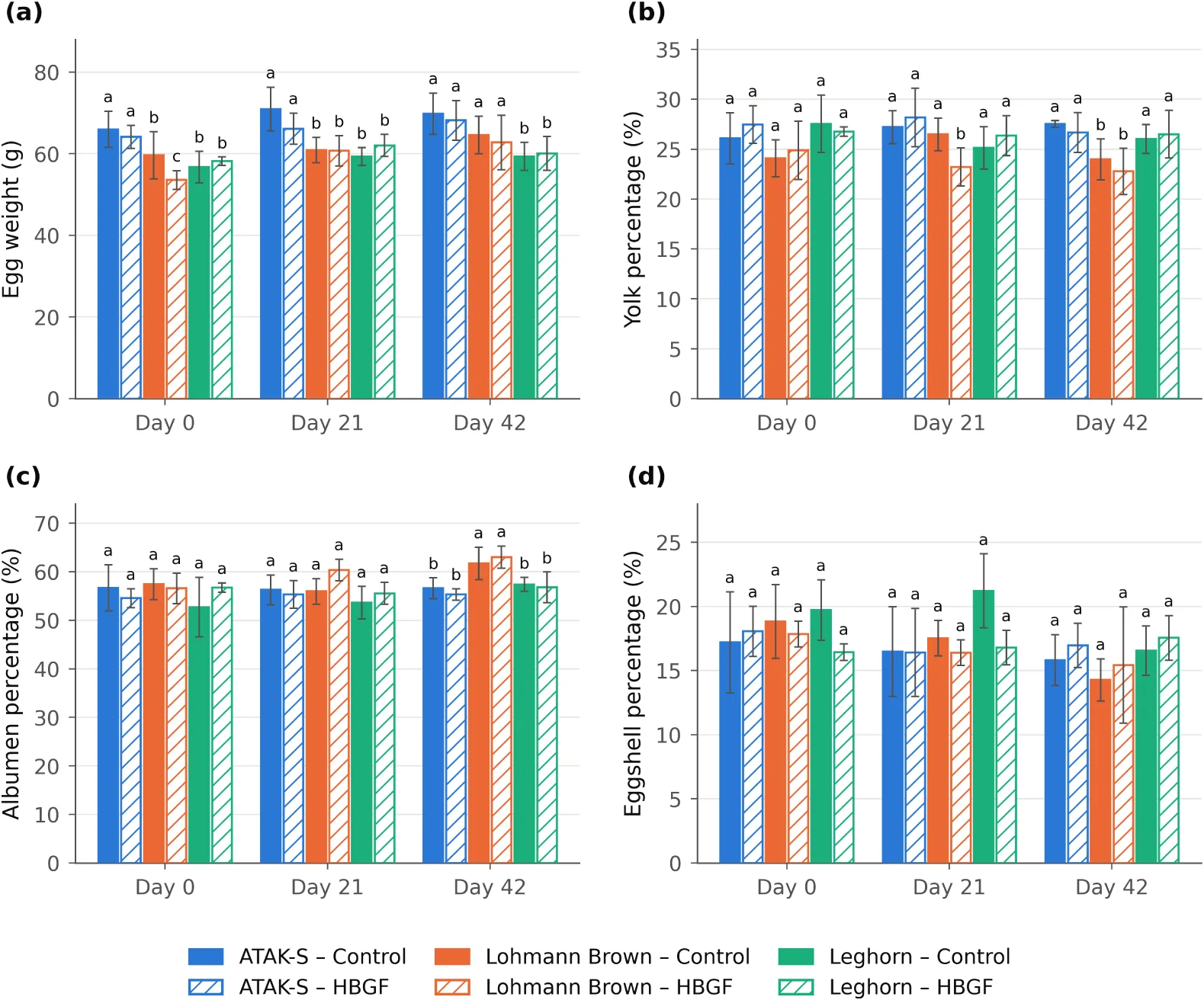
Egg content parameters of ATAK-S, Lohmann Brown, and Leghorn hens fed the control feed (filled bars) or the control feed plus hydroponic barley green fodder (HBGF; hatched bars) on days 0, 21, and 42: (a) egg weight, (b) yolk percentage, (c) albumen percentage, (d) eggshell percentage. Bars show means of four replicate subgroups; error bars show the standard deviation. Within each day, bars without a common letter differ (Duncan’s test, *p* < 0.05); all bars carry the same letter when no effect was significant in the two-way ANOVA for that day.

### 3.1 Egg Content Parameters

#### 3.1.1 Egg Weight

Egg weight differed among genotypes on every sampling day (*p* < 0.001 on days 0 and 21; *p* < 0.01 on day 42), whereas HBGF and the genotype × feed interaction had no effect on any day (Tables S1, S2 and S3; Figure 1a). ATAK-S hens laid the heaviest eggs throughout (64–71 g); Lohmann Brown (54–65 g) and Leghorn (57–62 g) laid lighter eggs on days 0 and 21, and on day 42, Lohmann Brown (63–65 g) no longer differed from ATAK-S, whereas Leghorn (59–60 g) remained lighter. This ranking was already present on day 0 and did not change during the trial, which points to genotype rather than feeding as the main driver. Egg weight is one of the most heritable egg traits (*h* ^2^ ≈ 0.6),^26^ and differences in egg weight between commercial hybrids and local or purebred genotypes have been reported under different housing conditions.^28,29^ The heavier eggs of ATAK-S agree with the free-range comparison of Sözcü et al.,^31^ in which ATAK-S laid heavier eggs than Atabey; Aygün et al.,^32^ on the other hand, reported lighter eggs for ATAK-S than for Lohmann Brown between 21 and 44 weeks of age. Differences in hen age and in feed intake under free-range conditions^32^ probably explain this discrepancy.

The lack of a feeding effect is consistent with the physiology of egg formation. Egg weight is determined largely by the mass of yolk deposited during the 7–10 days of rapid follicular growth before ovulation and by the amount of albumen secreted in the magnum, processes that are under endocrine control and respond to the overall energy and protein supply rather than to a fresh green supplement.^36,39^ Hydroponic barley is about 85–90% water and supplies little energy or protein per kilogram as fed^2,9^ (in dairy cows, for example, 15 kg of fresh hydroponic maize fodder supplied only about 0.6 kg of dry matter^40^), so when the basal diet is nutritionally complete, the extra nutrient intake from HBGF is small. Sandilands et al.^12^ likewise found no effect of up to 45 g/day of sprouted barley on egg weight in Lohmann Brown hens over 6 weeks, and hydroponic barley sprouts did not change egg weight in laying quail.^11^ Byproducts with a larger nutrient contribution can shift egg weight in either direction: dried fruit pomace reduced egg weight,^15^ whereas a multienzyme and probiotic combination had modest effects^41^ and probiotics did not change egg weight in a meta-analysis of 47 trials,^42^ which suggests that the outcome depends on the nutrient density of the additive rather than on its plant origin. Positive effects of hydroponic fodder on digestibility, growth, or milk yield in ruminants^13,14,40,43^ cannot be extrapolated to hens because of their different digestive physiology. Egg weight is governed mainly by strain, hen age, and the overall adequacy of the feed;^44^ the 42-day period used here is sufficient to reveal short-term effects but not long-term adaptations, and the equal metabolizable energy and crude protein supply of the basal feed in both groups explains the absence of a feeding effect.

#### 3.1.2 Albumen Percentage

Albumen percentage was not affected by genotype, HBGF, or their interaction on day 0, tended to differ among genotypes on day 21 (*p* = 0.052), and differed clearly on day 42 (*p* < 0.001), with no diet effect or interaction at any time (Tables S1, S2 and S3; Figure 1c). On day 42, Lohmann Brown hens had a higher albumen percentage (61.7–63.0%) than ATAK-S (55.4–56.6%) and Leghorn (56.8–57.4%), and the same tendency was visible on day 21 (60.4% in the Lohmann Brown HBGF group). A higher albumen proportion in Lohmann Brown is consistent with the intensive selection of commercial layer hybrids for egg output and egg quality^45^ and with genetic studies showing that albumen weight is highly heritable and genetically correlated with egg weight.^26,46^ Because yolk and albumen proportions are complementary, the higher albumen share of Lohmann Brown mirrors its lower yolk percentage (Section 3.1.4). That the genotype difference became detectable only as the trial progressed suggests that it developed with the advancing laying period rather than being present from the outset.

The absence of a diet effect is consistent with the view that internal egg quality is governed mainly by strain, hen age, and the overall adequacy of the diet.^44^ Albumen synthesis in the magnum depends on an adequate supply of crude protein (16–18%) and of the sulfur amino acids methionine and cysteine;^47^ HBGF, with a moderate protein concentration on a dry-matter basis and a very low dry-matter content as fed, adds little to this supply.^9,40^ Cufadar et al.^22^ showed that replacing soybean oil with glycerol did not alter egg weight or egg quality traits, only the yolk fatty acid profile. Antioxidant supplements such as organic selenium mainly preserve albumen and yolk quality during storage^48^ rather than increasing protein deposition, and although sprouted barley supplies phenolic compounds^6^ and green shoots supply carotenoids,^7,8^ no such effect on albumen proportion was detected here. Hens with access to vegetated range likewise showed unchanged albumen traits in the study of Kop-Bozbay et al.^49^ Environmental stressors such as heat depress egg weight and shell quality,^50^ but these were unlikely to differ between groups in the present trial. Taken together, genotype, not the fodder, determined albumen percentage in the present study.

#### 3.1.3 Eggshell Percentage

Eggshell percentage was not affected by genotype, HBGF, or their interaction on any sampling day (*p* > 0.05; Tables S1, S2 and S3; Figure 1d). Group means ranged from 14.3% to 21.2%, with the highest single values in the Leghorn control group on days 0 and 21 (19.7% and 21.2%) and in the Leghorn HBGF group on day 42 (17.6%), but the within-group variation was large relative to these differences. Notably, ATAK-S did not exhibit a greater shell percentage than the other genotypes despite having numerically the thickest shells (Section 3.2.1); this is because shell percentage is calculated in relation to egg weight, and the heavier eggs of ATAK-S reduce the shell ratio.

Eggshell formation takes about 18–20 h in the shell gland and depends on the supply of calcium and carbonate through uterine ion transporters, on medullary bone turnover, and on the hormonal control of these processes;^51–54^ differences among genotypes in shell quality are well documented,^55^ although shell traits are also strongly affected by environmental factors such as housing systems.^55,56^ Shell percentage is closely related to shell strength and breakage resistance.^44^ Shell traits respond to supplemental minerals mainly when the basal diet is limiting,^25^ and responses to trace-mineral sources are not always consistent;^57^ in hens fed an adequate feed, additional green forage has not been shown to improve shell deposition, and hydroponic barley did not affect shell traits in quail^11^ or shell thickness in hens.^12^ Some fruit-pomace supplements slightly reduced shell quality,^15^ but such effects were not seen with HBGF here. The absence of a genotype effect on shell percentage contrasts with the higher shell strength reported for commercial hybrids by Aygün et al.,^32^ illustrating that shell percentage, thickness, and breaking strength are related but distinct traits,^44^ and that with four replicate subgroups per group, the present trial had limited power to detect small differences in shell proportion.

#### 3.1.4 Yolk Percentage

Yolk percentage did not differ among the six groups on day 0 (genotype *p* = 0.061), but genotype had a significant effect on day 21 (*p* < 0.05) and day 42 (*p* < 0.01), while HBGF and the interaction had no effect at any time (Tables S1, S2 and S3; Figure 1b). ATAK-S (26.7–27.5%) and Leghorn (26.0–26.5%) had higher yolk percentages than Lohmann Brown (22.8–24.0%) on day 42, and on day 21, the Lohmann Brown HBGF group (23.2%) was lower than all other groups. The yolk supplies most of the energy, lipids, fat-soluble vitamins, and minerals of the egg,^58^ diet feeding, so this difference is nutritionally relevant. A lower yolk share in Lohmann Brown is the counterpart of its higher albumen proportion and is consistent with the strong positive genetic correlation between egg weight and albumen weight;^26,46^ comparable differences in the yolk-to-albumen ratio between commercial hybrids and purebred or local genotypes have been described previously.^28,29^ Yolk deposition proceeds over 7–10 days of rapid follicular growth under endocrine control,^39^ and its share of egg weight is not readily changed by a fresh forage supplement of low nutrient density when the basal diet is adequate, in line with the small effects of short-term supplements reported previously.^15,41^

### 3.2 Exterior Egg Quality Parameters

#### 3.2.1 Eggshell Thickness

Eggshell thickness did not differ among genotypes on any sampling day (*p* = 0.20–0.27), and no genotype × feed interaction was detected (Tables S1, S2 and S3; Figure 2b). The only significant term was a feeding effect on day 0 (*p* < 0.05), when the groups later assigned to HBGF had thinner shells than the control groups (5.82, 4.94, and 5.03 vs 6.03, 5.74, and 5.98 in ATAK-S, Lohmann Brown, and Leghorn, respectively); as this difference existed before the fodder was fed, it reflects initial group heterogeneity and disappeared by day 21. ATAK-S shells were numerically the thickest on all 3 days (6.03, 6.31, and 6.33 in the control groups), which agrees in direction with Sözcü et al.,^31^ who reported thicker and stronger shells in ATAK-S than in Atabey under free-range conditions, but the difference did not reach significance with four replicate subgroups per group. Shell thickness is moderately heritable (*h* ^2^ ≈ 0.34)^26^ and reflects genetic differences in intestinal calcium absorption, medullary bone mobilization, and carbonic anhydrase activity in the shell gland;^51,54^ genetic selection has improved shell strength across generations,^45^ although shell quality traits are also strongly influenced by environmental factors such as housing and nutrition.^55^

**Figure 2:**
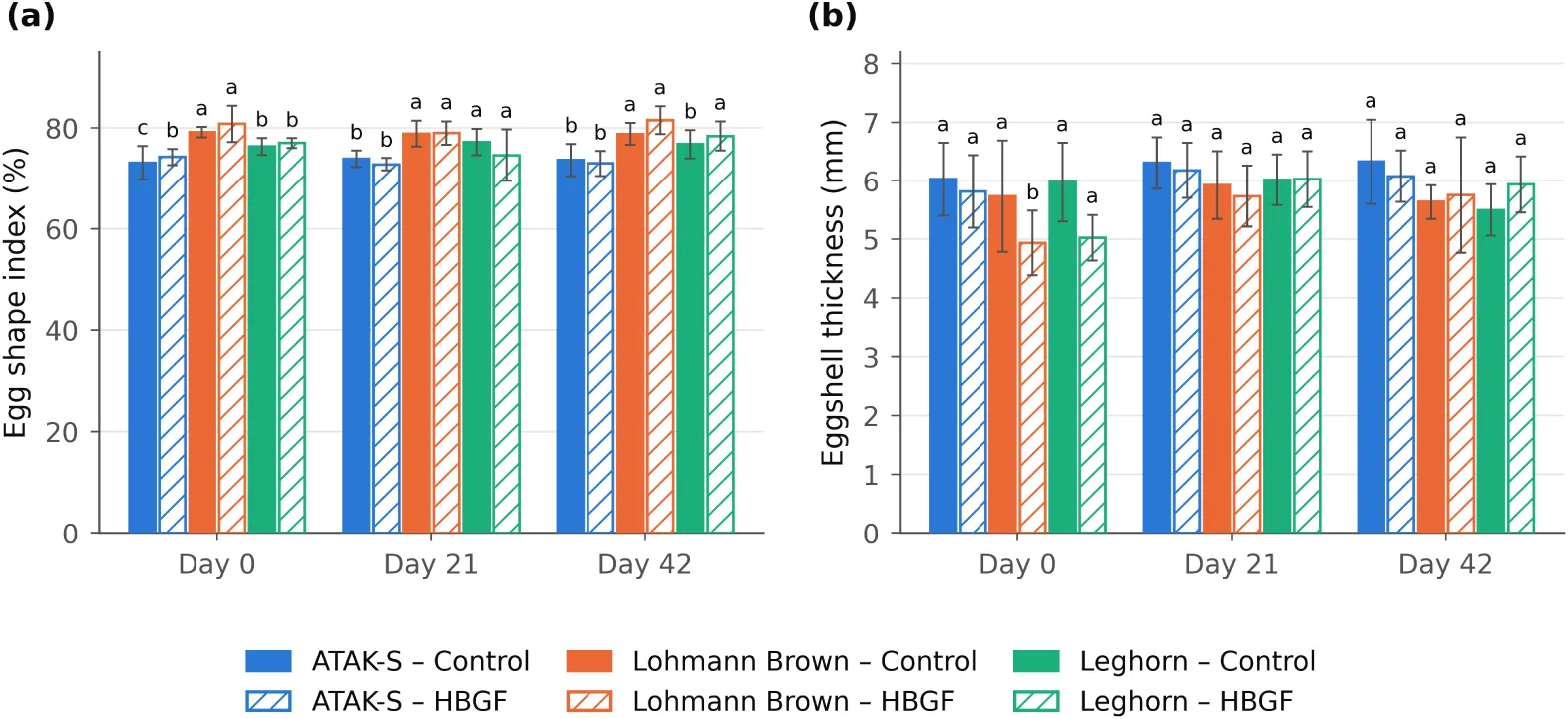
Exterior egg quality parameters of ATAK-S, Lohmann Brown, and Leghorn hens fed the control feed (filled bars) or the control feed plus HBGF (hatched bars) on days 0, 21, and 42: (a) egg shape index, (b) eggshell thickness. Bars show means of four replicate subgroups; error bars show the standard deviation. Within each day, bars without a common letter differ (Duncan’s test, *p* < 0.05); all bars carry the same letter when no effect was significant in the two-way ANOVA for that day.

Shell quality responds to feed mainly when calcium (3.5–4.0% of the diet), available phosphorus (0.3–0.4%), or vitamin D_3_ are limiting,^47^ and phosphorus can be reduced considerably without loss of performance when phytase is added;^59^ with an adequate basal diet, the vitamins and trace minerals supplied by HBGF are unlikely to increase calcification further, and hydroponic barley sprouts did not change shell thickness in quail.^11^ The present result agrees with Sandilands et al.,^12^ who found no effect of sprouted barley on shell thickness in Lohmann Brown hens, although they recorded transient decreases in shell breaking strength in some supplemented groups (15 and 45 g/day on day 21; 15 g/day on day 42); shell thickness and mechanical strength are related but not interchangeable measures.^44^ Feeding effects on shell traits in the literature are generally conditional: dietary zinc improved eggshell quality in broiler breeders, particularly under high environmental temperatures,^23^ and fava beans low in vicine and convicine had no detrimental effect on egg quality.^17^ Because shell quality declines with hen age as shell gland function decreases,^60^ HBGF might be more useful in older flocks or when the basal feed is marginal in calcium; in the mature hens on a complete feed used here, no such benefit was detectable.

#### 3.2.2 Egg Shape Index

The egg shape index differed among genotypes on all days (*p* < 0.01 on day 21; *p* < 0.001 on days 0 and 42) and was unaffected by HBGF or by the interaction (Tables S1, S2 and S3; Figure 2a). Lohmann Brown eggs were the roundest (78.8–81.5%) and ATAK-S eggs the most elongated (72.8–74.3%), with Leghorn intermediate (74.6–78.4%); on day 21, both Leghorn groups, and on day 42, only the Leghorn HBGF group, did not differ from Lohmann Brown. Egg shape is related to shell strength,^61^ and shape-index values of about 72–76% are commonly regarded as desirable for shell strength and packing; ATAK-S fell within this range, whereas Lohmann Brown was above it. Egg shape is fixed early in oviduct transit, in the magnum and isthmus, by oviduct anatomy, peristalsis, and egg rotation, and is largely genetically determined;^39^ it is not expected to respond to short-term feed changes. The stability of the index over the 42-day period confirms that this trait is homeostatic, in agreement with the unchanged shape index reported when hydroponic barley sprouts were fed to laying quail.^11^

### 3.3 Interior Egg Quality Parameters

#### 3.3.1 Yolk Color

Yolk color did not differ among genotypes on any sampling day and was not affected by HBGF or by the genotype × feed interaction on day 21 or day 42 (Tables S2 and S3; Figure 3a). On day 0, a feed effect (*p* < 0.05) and a genotype ×feed interaction (*p* < 0.01) were detected, owing to the lower score of the Lohmann Brown control group (12.03 vs 12.97–14.02 in the other groups); as these differences preceded the feeding treatment, they reflect initial group heterogeneity (Table S1). Fan scores were high in all groups (10.8–14.0), as expected for hens with access to range, and decreased in all groups from day 0 (12.0–14.0) to days 21 and 42 (10.8–12.0). On day 42, the HBGF groups were numerically slightly higher than their controls (ATAK-S 11.34 vs 11.25; Lohmann Brown 12.00 vs 11.61; Leghorn 11.67 vs 11.06), but none of these differences was significant.

**Figure 3:**
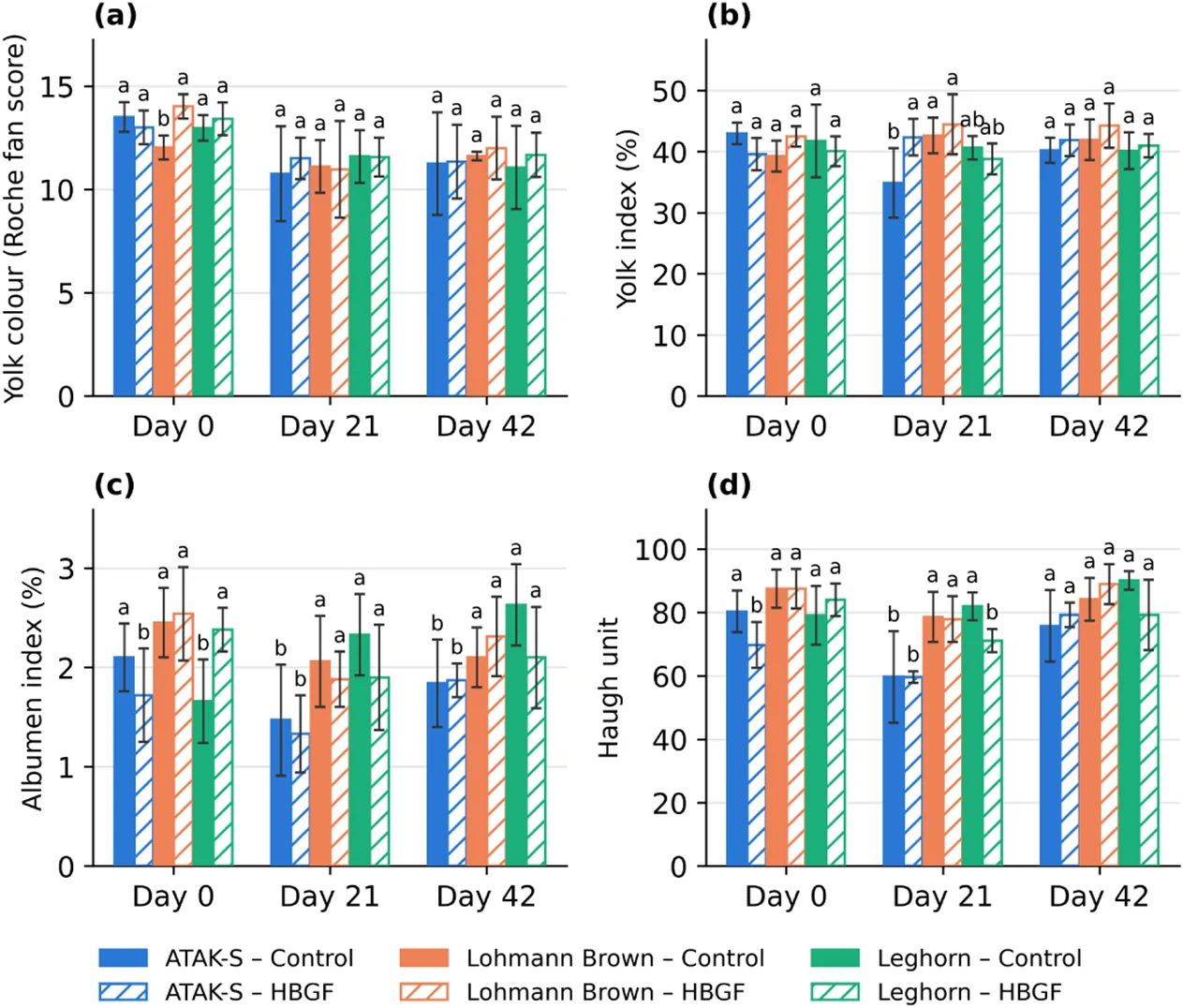
Interior egg quality parameters of ATAK-S, Lohmann Brown, and Leghorn hens fed the control (filled bars) or the control feed plus HBGF (hatched bars) on days 0, 21, and 42: (a) yolk color (Roche fan score), (b) yolk index, (c) albumen index, (d) Haugh unit. Bars show means of four replicate subgroups; error bars show the standard deviation. Within each day, bars without a common letter differ (Duncan’s test, *p* < 0.05); all bars carry the same letter when no effect was significant in the two-way ANOVA for that day.

Yolk color depends on dietary xanthophylls (mainly lutein and zeaxanthin) that are absorbed, carried in lipoproteins, and deposited in the developing follicles;^62,63^ because pigment accumulates during the final phase of follicular growth,^63^ a change in dietary pigment supply takes about 2–3 weeks to become fully visible in the yolk, so the 42-day period used here was long enough for a response to appear. Green plant material is a rich xanthophyll source: lucerne, marigold, and tomato raised fan scores markedly in Japanese quail,^62^ colored carrot forage increased yolk carotenoids within a short feeding period,^64^ and pasture or forage access more than doubles yolk lutein compared with cage systems.^65,66^ The absence of an HBGF effect is nevertheless consistent with the two previous studies on sprouted barley, in which neither Sandilands et al.^12^ in Lohmann Brown hens nor Abouelezz et al.^11^ in laying quail detected a change in yolk color. Two factors probably explain this. First, although green shoots and microgreens contain chlorophyll and carotenoids,^7,8^ the pigment supply of HBGF per kilogram as fed is low because of its high water content. Second, all hens in the present trial had access to range, and the high fan scores of the control groups (11–14) suggest that carotenoid intake from the range vegetation was already sufficient to pigment the yolks strongly, leaving little room for an additional response; under such conditions yolk color is governed mainly by the availability of range vegetation, which may also explain the parallel decline of the scores in all groups between day 0 and day 42. Genotype differences in yolk pigmentation have been reported under free-range conditions, with Lohmann Brown showing the most intensely colored yolks among three genotypes,^32^ but no genotype effect was detected here. Yolk color is a trait that feed can modify,^44,67^ and HBGF might contribute to pigmentation in indoor flocks without access to green forage, but such a benefit could not be demonstrated in the present free-range system.

#### 3.3.2 Yolk Index

Yolk index was not affected by HBGF or by the genotype × feed interaction on any sampling day, and genotype had an effect on day 21 only (*p* < 0.05; Tables S1, S2 and S3; Figure 3b). On day 21, the ATAK-S control group had the lowest value (34.9%), which differed from the ATAK-S HBGF (42.4%) and both Lohmann Brown groups (42.7–44.5%), while the Leghorn groups (38.8–40.6%) were intermediate. On days 0 and 42, the six groups did not differ (39.3–43.0% and 40.1–44.3%, respectively), and no consistent change over the feeding period was observed. The yolk index, the ratio of yolk height to yolk diameter, is related to the strength of the vitelline membrane^68^ and is used as an indicator of internal quality and freshness.^44^ The membrane is a multilayer glycoprotein structure whose inner layer is formed during follicular development and whose outer layer is added in the infundibulum,^69^ and its structure and strength vary among species.^68^ The values obtained here (35–44%) are in the range reported for fresh eggs of ATAK-S (43.1%) and Atabey (44.4%) under free-range conditions,^31^ and the genotype difference reported between those two Turkish genotypes^31^ was not reproduced among ATAK-S, Lohmann Brown, and Leghorn in the present study except on day 21. Feeding effects on yolk index are usually small in fresh eggs and become evident mainly after storage,^48^ and hydroponic barley sprouts did not change the egg quality traits of laying quail,^11^ so the absence of an HBGF effect over 42 days is consistent with the literature. The stability of the yolk index across the trial also indicates that vitelline membrane quality was not compromised by either feeding under the free-range conditions used.

#### 3.3.3 Albumen Index

Albumen index was influenced by genotype on every sampling day (*p* < 0.05), whereas HBGF had no effect; a genotype × feed interaction was detected on day 0 only (*p* < 0.05), before the feeds s diverged (Tables S1, S2 and S3; Figure 3c). On days 21 and 42, ATAK-S hens had the lowest albumen index (1.33–1.87%), and both Lohmann Brown (1.88–2.31%) and Leghorn (1.90–2.63%) were higher than ATAK-S but did not differ from each other; on day 0, the interaction reflected the low values of the ATAK-S HBGF (1.72%) and Leghorn control (1.66%) groups. Albumen height is moderately heritable (*h* ^2^ ≈ 0.5),^26^ and lower albumen quality in ATAK-S than in white-egg genotypes has been reported before.^31^ Sprouted or green forage supplements generally have little effect on albumen quality,^11,12,49^ in line with the limited effects of other feed additivs,^15,41^ and the present data give no indication that HBGF altered albumen index in any genotype.

#### 3.3.4 Haugh Unit

Haugh unit followed the same pattern as the albumen index. Genotype had a significant effect on day 0 (*p* < 0.01) and day 21 (*p* < 0.001), when Lohmann Brown (77.9–87.4) and Leghorn (71.1–84.0) had higher values, i.e., better albumen quality, than ATAK-S (59.6–80.3; on day 0, the difference was confined to the ATAK-S HBGF group, 69.7, whereas on day 21, both ATAK-S groups, 59.6–59.7, were lower); on day 42, the genotype effect was no longer significant (*p* = 0.073), although ATAK-S still had the numerically lowest values (75.7–79.2 vs 79.2–90.1) (Tables S1, S2 and S3; Figure 3d). Neither HBGF nor the genotype × feed interaction affected the Haugh unit on any day. Haugh unit is the standard measure of albumen freshness^37,70^ and is largely determined by genotype and hen age;^26,44^ lower Haugh units in ATAK-S than in Lohmann Brown and in white-egg genotypes have been reported under free-range conditions.^31,32^ Green fodder or range access usually leaves the Haugh unit unchanged,^49^ and sprouted barley did not affect it in quail.^11^ The present results therefore do not support any effect of HBGF on albumen quality, and confirm genotype as its main determinant.

### 3.4 Proximate Composition

The dry matter, crude protein, crude fat, and ash contents of egg white and yolk are given in Tables 2 and 3, respectively. Egg percentage (white versus yolk) was the dominant source of variation on every sampling day, although the two egg percentages were analyzed separately and this difference was not tested statistically. Yolk contained three to four times more dry matter than white (34.16–61.10% vs 12.10–14.76%), in accordance with the known composition of the egg, in which the yolk contains about 50% solids and the albumen 11–13%.^58,71^ Egg-white dry matter content declined slightly over the feeding period in ATAK-S and Leghorn (e.g., ATAK-S control: 14.76 → 13.34 → 13.18%; *p* < 0.05) but not in Lohmann Brown, whereas yolk dry matter content fluctuated without a consistent trend.

**Table 2:** Dry Matter, Crude Protein, and Ash Contents (%) of Egg White from Three Laying-Hen Genotypes Fed with or without Hydroponic Barley Green Fodder over the Feeding Period (wet basis, Mean ± SD)^a^

Feeding period	Genotype	Treatment	Dry matter content (%)	Crude protein content (%)	Ash content (%)
Day 0	ATAK-S	Control	14.76 ± 1.06^aA^	12.69 ± 0.09^abA^	0.50 ± 0.06^Aa^
		HBGF	13.64 ± 0.21^aB^	11.61 ± 0.09^bA^	0.58 ± 0.04^Aa^
	Lohmann Brown	Control	13.14 ± 0.90^aA^	10.95 ± 0.08^cA^	0.49 ± 0.06^Aa^
		HBGF	13.55 ± 0.83^aA^	13.00 ± 0.10^aA^	0.43 ± 0.04^Ba^
	Leghorn	Control	14.29 ± 0.57^aA^	13.48 ± 0.10^aA^	0.52 ± 0.01^Aa^
		HBGF	14.30 ± 0.36^aA^	11.95 ± 0.09^bcA^	0.49 ± 0.05^Aa^
Day 21	ATAK-S	Control	13.34 ± 0.87^bA^	10.13 ± 0.08^cB^	0.43 ± 0.03^Ab^
		HBGF	12.87 ± 0.56^abB^	12.25 ± 0.09^aA^	0.39 ± 0.05^Bb^
	Lohmann Brown	Control	13.02 ± 0.62^aA^	8.14 ± 0.06^dB^	0.38 ± 0.03^Bb^
		HBGF	13.48 ± 0.37^aA^	13.08 ± 0.10^aA^	0.35 ± 0.02^Bb^
	Leghorn	Control	13.39 ± 0.35^abA^	11.00 ± 0.08^bB^	0.35 ± 0.03^Bb^
		HBGF	13.33 ± 0.75^abA^	10.75 ± 0.08^bA^	0.42 ± 0.02^Ab^
Day 42	ATAK-S	Control	13.18 ± 0.92^bA^	10.18 ± 0.08^bB^	0.45 ± 0.04^Aa^
		HBGF	12.10 ± 0.49^bB^	10.65 ± 0.08^bB^	0.47 ± 0.07^Aa^
	Lohmann Brown	Control	13.25 ± 1.13^aA^	10.81 ± 0.08^bcA^	0.57 ± 0.07^Aa^
		HBGF	13.93 ± 0.88^aA^	10.57 ± 0.08^bB^	0.39 ± 0.05^Bb^
	Leghorn	Control	12.96 ± 0.34^bB^	10.98 ± 0.08^bB^	0.44 ± 0.05^Ba^
		HBGF	12.25 ± 0.16^bB^	11.50 ± 0.09^abA^	0.37 ± 0.07^Bb^

a **Note:** Values are expressed as mean ± SD. Different **lowercase superscript letters** within the same genotype × treatment combination indicate significant differences among feeding periods (Day 0, Day 21, and Day 42). Different **uppercase superscript letters** within the same feeding period and egg percentage indicate significant differences among the six genotype × treatment combinations (*p* < 0.05). Egg white and egg yolk were analyzed separately. Crude fat content was not determined in egg white because of its negligible level.

**Table 3:** Dry Matter, Crude Protein, Crude Fat, and Ash Contents (%) of Egg Yolk from Three Laying-Hen Genotypes Fed with or without Hydroponic Barley Green Fodder over the Feeding Period (wet basis, Mean ± SD)^a^

Feeding period	Genotype	Treatment	Dry matter content (%)	Crude protein content (%)	Crude fat content (%)	Ash content (%)
Day 0	ATAK-S	Control	61.10 ± 9.91^aA^	14.61 ± 0.11^cB^	54.41 ± 1.12^aB^	1.62 ± 0.02^Aa^
		HBGF	49.73 ± 1.84^bB^	14.46 ± 0.11^cB^	41.03 ± 1.05^cD^	1.36 ± 0.01^Ba^
	Lohmann Brown	Control	47.09 ± 4.64^bB^	15.17 ± 0.11^bB^	52.96 ± 1.16^aBC^	1.51 ± 0.02^Aa^
		HBGF	46.26 ± 4.66^bB^	15.62 ± 0.12^bA^	53.94 ± 1.18^bB^	1.40 ± 0.08^Ba^
	Leghorn	Control	45.77 ± 5.11^bB^	16.39 ± 0.12^aA^	64.81 ± 1.35^aA^	1.36 ± 0.01^Ba^
		HBGF	58.17 ± 2.14^aA^	16.68 ± 0.12^aA^	50.87 ± 1.20^aC^	1.31 ± 0.10^Ba^
Day 21	ATAK-S	Control	47.39 ± 2.75^bB^	16.01 ± 0.12^aA^	51.87 ± 1.24^bD^	1.54 ± 0.06^Aa^
		HBGF	49.14 ± 0.95^bB^	16.23 ± 0.12^aA^	73.89 ± 1.42^aA^	1.07 ± 0.46^Bb^
	Lohmann Brown	Control	34.16 ± 5.20^cB^	16.66 ± 0.12^aA^	44.00 ± 1.11^bF^	1.23 ± 0.28^Bb^
		HBGF	47.77 ± 1.21^bB^	15.96 ± 0.12^aA^	59.95 ± 1.21^aB^	1.57 ± 0.10^Aa^
	Leghorn	Control	43.34 ± 9.51^bB^	15.18 ± 0.11^bB^	56.05 ± 1.27^bC^	1.61 ± 0.01^Aa^
		HBGF	48.89 ± 4.89^bB^	16.19 ± 0.12^aA^	46.80 ± 1.14^bE^	1.50 ± 0.06^Aa^
Day 42	ATAK-S	Control	50.95 ± 0.70^bB^	16.44 ± 0.12^aA^	48.92 ± 1.18^cD^	1.61 ± 0.08^Aa^
		HBGF	52.29 ± 1.91^aA^	15.62 ± 0.11^aA^	68.60 ± 1.36^bA^	1.54 ± 0.10^Aa^
	Lohmann Brown	Control	51.98 ± 2.13^aA^	16.25 ± 0.12^aA^	41.28 ± 1.09^cF^	1.63 ± 0.02^Aa^
		HBGF	50.11 ± 0.41^aA^	15.62 ± 0.11^aA^	57.63 ± 1.19^aB^	0.56 ± 0.85^Bb^
	Leghorn	Control	51.11 ± 1.71^aA^	16.09 ± 0.12^aA^	53.74 ± 1.22^bC^	1.61 ± 0.03^Aa^
		HBGF	51.01 ± 0.31^bA^	15.76 ± 0.11^aB^	43.95 ± 1.10^cE^	1.48 ± 0.16^Aa^

a **Note:** Values are expressed as mean ± SD. Different **lowercase superscript letters** within the same genotype × treatment combination indicate significant differences among feeding periods (Day 0, Day 21, and Day 42). Different **uppercase superscript letters** within the same feeding period and egg percentage indicate significant differences among the six genotype × treatment combinations (*p* < 0.05). Egg white and egg yolk were analyzed separately.

Crude protein content followed the same egg-percentage-driven pattern: yolk protein (14.46–16.68%) exceeded white protein (8.14–13.48%) on a fresh-weight basis, close to the reported values of about 16% and 10–11%.^58^ The clearest feeding effect was observed in egg white on day 21, when HBGF hens had higher albumen protein than controls in Lohmann Brown (13.08 vs 8.14%) and ATAK-S (12.25 vs 10.13%; *p* < 0.05); no such difference was seen in Leghorn or on day 42, and yolk protein did not differ consistently between feedings. It should be noted, however, that the Lohmann Brown HBGF group already had a higher albumen protein content than its control on day 0 (13.00 vs 10.95%; *p* < 0.05), before the fodder was fed, so part of the day 21 difference in this genotype reflects initial group heterogeneity. Germination raises the crude protein^2,5,6^ and amino acid^5^ content of barley, but given the small dry-matter intake from HBGF, a direct nutritional explanation for an increase of 2–5 percentage points in albumen protein is unlikely; the transient nature of the effect suggests that it should be confirmed before it is attributed to the fodder.

Crude fat content was determined only in the yolk; it ranged from 41.03% to 73.89%. HBGF groups had higher yolk fat than their controls in ATAK-S (73.89 vs 51.87% on day 21; 68.60 vs 48.92% on day 42) and in Lohmann Brown (59.95 vs 44.00% on day 21; 57.63 vs 41.28% on day 42), whereas Leghorn HBGF yolks contained less fat than their controls (46.80 vs 56.05% on day 21; 43.95 vs 53.74% on day 42; all *p* < 0.05). These feeding-related differences should be interpreted with caution, because on day 0, the ATAK-S group later assigned to HBGF already had a lower yolk fat content than its control (41.03 vs 54.41%; *p* < 0.05) and the Leghorn HBGF group likewise had a lower yolk fat content than its control (50.87 vs 64.81%; *p* < 0.05). The total lipid content of the yolk is relatively stable and is not easily changed by diet,^72^ although its fatty acid profile responds readily to dietary lipids and to forage intake;^49,66,72,73^ sprouted wheat, for example, only marginally lowered the yolk n-6/n-3 ratio relative to a maize-based control, although it reduced yolk cholesterol.^74^ The genotype-dependent direction of the change suggests that differences between hybrids in the partitioning of dietary energy into yolk lipid, rather than a uniform effect of the fodder, underlie these results. The upper values (above 65% of dry matter) are high relative to the roughly 60–65% of yolk solids that lipids usually represent (about 31–33% lipid in a yolk containing about 50% solids)^58,71^ and merit reexamination.

Ash content again separated the two egg percentages: yolk ash content (0.56–1.63%) was about three times higher than white ash content (0.35–0.58%), in line with reported values of about 1.6–1.7% and 0.5–0.6%.^58^ In egg white, ash content decreased from day 0 to day 21 in all groups and partially recovered by day 42 in four of the six groups (e.g., ATAK-S HBGF: 0.58 → 0.39 → 0.47%; *p* < 0.05). HBGF did not increase white ash content consistently: within genotypes, the HBGF group had lower white ash content than its control in Lohmann Brown on days 0 and 42 (0.43 vs 0.49% and 0.39 vs 0.57%) and in ATAK-S on day 21 (0.39 vs 0.43%), higher white ash content only in Leghorn on day 21 (0.42 vs 0.35%; *p* < 0.05), and did not differ from its control in the remaining comparisons. Yolk ash content likewise showed no consistent feeding effect. Part of the variability among genotypes and days may stem from variation in the composition of the fodder itself, which changes with plant species, mixture, and growing conditions,^75^ and from the source and bioavailability of the minerals supplied.^25,76^ Overall, egg percentage was the principal determinant of proximate composition, whereas HBGF exerted secondary, genotype- and time-dependent effects that, given that analytical triplicates rather than replicate subgroups formed the unit of variance and that some feeding-related differences were already present on day 0, should be regarded as preliminary.

## 4 Conclusion

Genotype was the main factor determining egg quality and the proximate composition of eggs in this study, whereas supplementation with hydroponic barley green fodder had limited effects on hens fed a nutritionally complete feed ATAK-S hens laid the heaviest eggs, Lohmann Brown hens had the highest albumen percentage and egg shape index, and Lohmann Brown and Leghorn hens had higher albumen index and Haugh unit values than ATAK-S; eggshell thickness, eggshell percentage, yolk color, and yolk index did not differ consistently among genotypes. Proximate composition was governed by egg percentage, with the yolk consistently containing more dry matter, protein, fat, and ash than the albumen. No physical egg quality trait was affected by HBGF on days 21 or 42; in particular, yolk color, which was already intense in all groups under free-range conditions, and yolk index were not changed by the fodder. HBGF also induced genotype- and time-dependent changes in proximate composition, such as higher albumen protein content on day 21 in ATAK-S and Lohmann Brown and higher yolk fat content in ATAK-S and Lohmann Brown but lower yolk fat content in Leghorn, with no consistent effect on albumen ash content, which suggests that dietary nutrients are used differently by the three genotypes. Overall, genetics had a greater influence on egg quality and composition than short-term HBGF supplementation, and any benefit of HBGF for yolk pigmentation would have to be demonstrated in flocks without access to green forages. Longer feeding periods, defined inclusion levels, characterization of the fodder actually consumed, and larger replicate numbers are needed to establish the nutritional role of hydroponic green fodder in egg production.

## Supplementary Material



## Data Availability

The data sets generated and analyzed during this study are included in the Supporting Information accompanying this article.

## Notes

Egg quality parameters on days 0, 21, and 42 (Tables S1–S3) (PDF)
