# Supplementary material for: Effects of Hydroponic Barley Green Fodder Supplementation on Egg Quality and Proximate Composition in ATAK-S, Lohmann Brown, and Leghorn Laying Hens
Source: ACS Omega. 2026 Oct 20;11(43):67906–16. doi: 10.1021/acsomega.6c10822 (PMC13737906; doi:10.1021/acsomega.6c10822)
Supplement: Supplementary file 1 [file ao6c10822_si_001.pdf]

Supplementary Material

Effects of Hydroponic Barley Green Fodder Supplementation on Egg Quality and Proximate Composition in ATAK-S, Lohmann Brown, and Leghorn Laying Hens

Süleyman Hacısalihoğlu, Samiye Adal, Zeynep Nur Karakuş, Yasemin Çelebi, Gülşah Çalışkan Koç

Table S1. Effects of genotype (G), diet (T) and their interaction (G × T) on egg quality parameters of ATAK-S, Lohmann Brown and Leghorn hens on day 0 (baseline)

Parameters		ATAK-S Control	ATAK-S Trial	Lohmann Brown Control	Lohmann Brown Trial	Leghorn Control	Leghorn Trial	Genotype (G)	Treatment (T)	G × T
EC	**EW (g)**	66.01 ± 4.42[a]	64.14 ± 2.84[a]	59.65 ± 5.81[b]	53.59 ± 2.29[c]	56.76 ± 3.88[b]	58.21 ± 1.03[b]	***	ns	ns
	**YP (%)**	26.10 ± 2.56[a]	27.48 ± 1.88[a]	24.09 ± 1.85[a]	24.90 ± 2.92[a]	27.56 ± 2.89[a]	26.77 ± 0.47[a]	ns	ns	ns
	**AP (%)**	56.68 ± 4.75[a]	54.57 ± 1.95[a]	57.45 ± 3.16[a]	56.61 ± 3.14[a]	52.72 ± 6.13[a]	56.77 ± 0.94[a]	ns	ns	ns
	**EP (%)**	17.21 ± 3.93[a]	18.06 ± 1.95[a]	18.84 ± 2.88[a]	17.86 ± 1.01[a]	19.72 ± 2.35[a]	16.44 ± 0.65[a]	ns	ns	ns
EEQP	**ESI (%)**	73.09 ± 3.34[c]	74.28 ± 1.62[b]	79.19 ± 1.00[a]	80.83 ± 3.58[a]	76.35 ± 1.68[b]	77.02 ± 0.99[b]	***	ns	ns
	**ET (mm)**	6.03 ± 0.62[a]	5.82 ± 0.62[a]	5.74 ± 0.95[a]	4.94 ± 0.55[b]	5.98 ± 0.67[a]	5.03 ± 0.39[a]	ns	*	ns
IEQP	**YI (%)**	42.99 ± 1.76[a]	39.58 ± 2.64[a]	39.27 ± 2.51[a]	42.49 ± 1.66[a]	41.74 ± 5.95[a]	40.05 ± 2.44[a]	ns	ns	ns
	**YC**	13.50 ± 0.72[a]	13.00 ± 0.81[a]	12.03 ± 0.58[b]	14.02 ± 0.59[a]	12.97 ± 0.62[a]	13.41 ± 0.79[a]	ns	*	**
	**AI (%)**	2.10 ± 0.34[a]	1.72 ± 0.47[b]	2.45 ± 0.35[a]	2.54 ± 0.47[a]	1.66 ± 0.42[b]	2.38 ± 0.22[a]	*	ns	*
	**HU**	80.30 ± 6.58[a]	69.71 ± 7.25[b]	87.43 ± 6.04[a]	87.43 ± 6.19[a]	79.05 ± 9.31[a]	83.97 ± 5.13[a]	**	ns	ns

Different superscript letters (a–c) within a row indicate significant differences among the six genotype × diet means according to Duncan's multiple range test ($p < 0.05$). G = genotype effect, T = diet (HBGF) effect, G × T = genotype × diet interaction; * $p < 0.05$, ** $p < 0.01$, *** $p < 0.001$, ns = not significant. Trial = HBGF-supplemented group. EC: egg content parameters; EEQP: exterior egg quality parameters; IEQP: interior egg quality parameters; EW: egg weight; YP: yolk percentage; AP: albumen percentage; EP: eggshell percentage; ESI: egg shape index; ET: eggshell thickness; YC: yolk colour (Roche fan score); YI: yolk index; AI: albumen index; HU: Haugh unit.

Table S2. Effects of genotype (G), diet (T) and their interaction (G × T) on egg quality parameters of ATAK-S, Lohmann Brown and Leghorn hens on day 21

Parameters		ATAK-S Control	ATAK-S Trial	Lohmann Brown Control	Lohmann Brown Trial	Leghorn Control	Leghorn Trial	G	T	G×T
EC	**EW (g)**	70.93 ± 5.35[a]	66.16 ± 3.79[a]	60.93 ± 3.10[b]	60.76 ± 3.73[b]	59.37 ± 2.18[b]	62.04 ± 2.71[b]	***	ns	ns
	**YP (%)**	27.23 ± 1.65[a]	28.20 ± 2.93[a]	26.50 ± 1.63[a]	23.23 ± 1.92[b]	25.14 ± 2.13[a]	26.37 ± 2.00[a]	*	ns	ns
	**AP (%)**	56.28 ± 3.05[a]	55.38 ± 2.84[a]	55.98 ± 2.62[a]	60.38 ± 2.21[a]	53.63 ± 3.35[a]	55.57 ± 2.26[a]	ns	ns	ns
	**EP (%)**	16.49 ± 3.50[a]	16.42 ± 3.42[a]	17.53 ± 1.38[a]	16.40 ± 0.99[a]	21.22 ± 2.88[a]	16.80 ± 1.35[a]	ns	ns	ns
EEQP	**ESI (%)**	73.91 ± 1.64[b]	72.81 ± 1.22[b]	78.90 ± 2.58[a]	79.02 ± 2.32[a]	77.24 ± 2.60[a]	74.63 ± 5.09[a]	**	ns	ns
	**ET (mm)**	6.31 ± 0.44[a]	6.18 ± 0.47[a]	5.93 ± 0.58[a]	5.74 ± 0.52[a]	6.02 ± 0.43[a]	6.03 ± 0.48[a]	ns	ns	ns
IEQP	**YI (%)**	34.88 ± 5.68[b]	42.35 ± 3.00[a]	42.65 ± 2.91[a]	44.48 ± 4.93[a]	40.61 ± 1.92[ab]	38.81 ± 2.50[ab]	*	ns	ns
	**YC**	10.76 ± 2.29[a]	11.50 ± 1.01[a]	11.11 ± 1.27[a]	10.97 ± 2.34[a]	11.59 ± 1.27[a]	11.56 ± 0.94[a]	ns	ns	ns
	**AI (%)**	1.47 ± 0.56[b]	1.33 ± 0.39[b]	2.06 ± 0.46[a]	1.88 ± 0.28[a]	2.33 ± 0.41[a]	1.90 ± 0.53[a]	*	ns	ns
	**HU**	59.65 ± 14.43[b]	59.57 ± 1.83[b]	78.55 ± 7.92[a]	77.85 ± 7.24[a]	81.93 ± 4.39[a]	71.07 ± 3.67[a]	***	ns	ns

Different superscript letters (a–c) within a row indicate significant differences among the six genotype × diet means according to Duncan's multiple range test ($p < 0.05$). G = genotype effect, T = diet (HBGF) effect, G × T = genotype × diet interaction; * $p < 0.05$, ** $p < 0.01$, *** $p < 0.001$, ns = not significant. Trial = HBGF-supplemented group. EC: egg content parameters; EEQP: exterior egg quality parameters; IEQP: interior egg quality parameters; EW: egg weight; YP: yolk percentage; AP: albumen percentage; EP: eggshell percentage; ESI: egg shape index; ET: eggshell thickness; YC: yolk color (Roche fan score); YI: yolk index; AI: albumen index; HU: Haugh unit.

Table S3. Effects of genotype (G), diet (T) and their interaction (G × T) on egg quality parameters of ATAK-S, Lohmann Brown and Leghorn hens on day 42

Parameters		ATAK-S Control	ATAK-S Trial	Lohmann Brown Control	Lohmann Brown Trial	Leghorn Control	Leghorn Trial	G	T	G×T
EC	**EW (g)**	69.83 ± 5.06[a]	68.20 ± 4.83[a]	64.63 ± 4.59[a]	62.79 ± 6.68[a]	59.37 ± 3.43[b]	60.09 ± 4.12[b]	**	ns	ns
	**YP (%)**	27.54 ± 0.33[a]	26.69 ± 2.01[a]	23.99 ± 2.04[b]	22.79 ± 2.32[b]	26.03 ± 1.44[a]	26.51 ± 2.40[a]	**	ns	ns
	**AP (%)**	56.64 ± 2.16[b]	55.35 ± 1.17[b]	61.74 ± 3.31[a]	63.03 ± 2.30[a]	57.41 ± 1.44[b]	56.82 ± 3.14[b]	***	ns	ns
	**EP (%)**	15.83 ± 1.98[a]	16.97 ± 1.72[a]	14.28 ± 1.64[a]	15.43 ± 4.53[a]	16.57 ± 1.92[a]	17.56 ± 1.73[a]	ns	ns	ns
EEQP	**ESI (%)**	73.62 ± 3.23[b]	72.99 ± 2.46[b]	78.82 ± 2.15[a]	81.54 ± 2.77[a]	76.80 ± 2.80[b]	78.42 ± 2.88[a]	***	ns	ns
	**ET (mm)**	6.33 ± 0.72[a]	6.08 ± 0.44[a]	5.64 ± 0.29[a]	5.76 ± 0.99[a]	5.50 ± 0.44[a]	5.94 ± 0.48[a]	ns	ns	ns
IEQP	**YI (%)**	40.22 ± 2.06[a]	41.87 ± 2.59[a]	41.94 ± 3.32[a]	44.27 ± 3.62[a]	40.14 ± 3.00[a]	40.97 ± 1.91[a]	ns	ns	ns
	**YC**	11.25 ± 2.48[a]	11.34 ± 1.79[a]	11.61 ± 0.21[a]	12.00 ± 1.52[a]	11.06 ± 2.01[a]	11.67 ± 1.08[a]	ns	ns	ns
	**AI (%)**	1.84 ± 0.44[b]	1.87 ± 0.17[b]	2.10 ± 0.30[a]	2.31 ± 0.40[a]	2.63 ± 0.41[a]	2.10 ± 0.51[a]	*	ns	ns
	**HU**	75.72 ± 11.30[a]	79.17 ± 3.88[a]	84.10 ± 6.79[a]	88.89 ± 6.33[a]	90.06 ± 2.89[a]	79.20 ± 11.11[a]	ns	ns	ns

Different superscript letters (a–c) within a row indicate significant differences among the six genotype × diet means according to Duncan's multiple range test ($p < 0.05$). G = genotype effect, T = diet (HBGF) effect, G × T = genotype × diet interaction; * $p < 0.05$, ** $p < 0.01$, *** $p < 0.001$, ns = not significant. Trial = HBGF-supplemented group. EC: egg content parameters; EEQP: exterior egg quality parameters; IEQP: interior egg quality parameters; EW: egg weight; YP: yolk percentage; AP: albumen percentage; EP: eggshell percentage; ESI: egg shape index; ET: eggshell thickness; YC: yolk color (Roche fan score); YI: yolk index; AI: albumen index; HU: Haugh unit. Entries in blue differ from the originally submitted table.
